# Lessons learned from Café Scientifique public webinars on pregnancy and parenting in arthritis: perspectives from patients, clinicians, and researchers

**DOI:** 10.1186/s12919-025-00323-7

**Published:** 2025-04-08

**Authors:** Vienna Cheng, Preet Kang, Laurie Proulx, Neda Amiri, Mary A. De Vera

**Affiliations:** 1 Faculty of Pharmaceutical Sciences, University of British Columbia, 2405 Wesbrook Mall, Vancouver, BC V6T 1Z3 Canada; 2 Collaboration for Outcomes Research and Evaluation, Vancouver, BC Canada; 3 Arthritis Research Canada, Vancouver, BC Canada; 4 Canadian Arthritis Patient Alliance, Ottawa, ON Canada; 5 Division of Rheumatology, Department of Medicine, Faculty of Medicine, University of British Columbia, Vancouver, BC Canada; 6 Centre for Advancing Health Outcomes, Vancouver, BC Canada

**Keywords:** Pregnancy, Rheumatic disease, Patients, Parenting, Arthritis, Lived experience, Challenges

## Abstract

**Background:**

Arthritis is a group of chronic inflammatory conditions, including systemic lupus erythematosus and rheumatoid arthritis, that disproportionately impact females more than men, often during the childbearing years. Challenges with pregnancy and parenting continue to significantly impact patients and families living with arthritis, compounded by the historical lack of research that has hindered the ability to make informed family planning decisions.

**Objectives:**

In ongoing efforts to broadly translate our expanding research findings in this area, we held two public webinars on pregnancy and parenting in arthritis with the following goals: 1) to create forums for discussion among researchers, patients, and clinicians; 2) raise awareness on emerging issues requiring future research; and 3) provide evidence-based, practical advice for patients, caregivers and families.

**Summary:**

The first webinar on Pregnancy and Arthritis united perspectives from patients, clinicians and researchers. We learned firsthand challenges of navigating pregnancy with arthritis from patients of various backgrounds, including those who highlighted longstanding healthcare disparities. A rheumatologist specializing in pregnancy and reproductive health shared insights, concluding with the positive outlook that perinatal research is underway on emerging antirheumatic therapies. The second webinar on Parenting and Arthritis discussed evidence-based resources and strategies to support parents, integrating insights from researchers and clinicians.

**Conclusion:**

These webinars illuminated the profound impacts of arthritis on patients and families and revealed knowledge gaps for future research.

## Introduction

Arthritis is a group of chronic inflammatory conditions, including systemic lupus erythematosus and rheumatoid arthritis, that disproportionately impact females more than males, and often strike during reproductive years [1]. Biological processes during pregnancy, including endocrine and immune changes, account for clinical and therapeutic challenges in arthritis, as multiple aspects of pregnancy such as neonatal and maternal outcomes are influenced by autoimmunity. Pregnancy and parenting issues impact patients (and their caregivers and families) living arthritis and there are gaps in research that disempower families from making informed decisions.

## Objectives

Pregnancy and parenting in arthritis have historically been understudied. However, over the past decade, research, including patient-oriented studies conducted by our team, has generated evidence that better support patients with arthritis who are or planning to become pregnant. In ongoing efforts to broadly translate findings from this research, the goals of our public webinars on pregnancy and parenting in arthritis were to: 1) create forums for discussion among researchers, patients, and clinicians; 2) raise awareness and identify emerging issues that can be addressed with future research; and 3) provide evidence-based, practical advice for patients and families.

## Webinars

We held two public “Café Scientifique” webinars on: 1) Pregnancy and arthritis (May 2024) and 2) Parenting and arthritis (June 2024)—with support from the Canadian Institute of Health Research. The webinars were hosted by Canadian Arthritis Patient Alliance (CAPA) with presenters and organizers affiliated with CAPA, Arthritis Research Canada, and the University of British Columbia (UBC).

Each 90-min Zoom webinar featured panel presentations, interactive components (chat, polls, etc.), and a question-and-answer period. Webinars were moderated by an arthritis community leader (LP) who has lived experience with rheumatoid arthritis and multiple pregnancies. Presenters for each webinar included patients, clinicians, and researchers representing a wide range of experience and perspectives, reflecting values of engagement and diversity. Both webinars were recorded and made available on the CAPA YouTube Channel. Presenters also contributed blogs related to their topics, shared on the CAPA website. Table 1 provides a summary of topics and presenters for each webinar.

**Table 1:** Programmes for the Café Scientifique Webinars

**Pregnancy and arthritis webinar (May 2024)**		
Moderator		
Introduction & land acknowledgement	Laurie Proulx	Patient
Speakers		
The painful road to becoming pregnant with rheumatoid arthritis	Cristina Montoya	Patient
Pregnancy and rheumatic diseases clinic	Dr. Neda Amiri	Clinician (rheumatologist)
Health inequities among black women with arthritis	Nazret Russon and Shani Ellis-Alman	Patients
Arthritis and Pregnancy Research Updates	Dr. Mary De Vera	Researcher
Updated Evidence on Targeted Therapies in Pregnancy	Dr. Vienna Cheng	Clinician (pharmacist)/Researcher
**Parenting and arthritis webinar (June 2024)**		
Moderator		
Introduction & land acknowledgement	Laurie Proulx	Patient
Speakers		
She walks with me: the urban indigenous doula project	Ashley Hayward	Researcher
Tips for parenting with arthritis	Mariah Leach^a^	Patient
Parenting and arthritis: evidence-based advice	Dr. Catherine Backman	Clinician (occupational therapist)/Researcher

^a^Unable to present

### Perspectives shared on pregnancy and arthritis

Our first webinar united diverse perspectives on Pregnancy and Arthritis from patients, clinicians and researchers. We heard from firsthand challenges of navigating pregnancy with arthritis amongst various backgrounds, paired with viewpoints from a rheumatologist specializing in pregnancy and reproductive health.

Embarking on the pregnancy journey often comes with anticipation. Yet, for the patient panelist living with rheumatoid arthritis, the road to delivering her first baby was filled with feelings of being dismissed from various physicians regarding her intentions to start a family and when pregnancy occurred. When she was finally referred to a pregnancy and arthritis specialist, she was grateful to have sought out community supports for advice on preparing for appointments. However, after delivering her baby, she found herself in a place of feeling abandoned by the healthcare system. While navigating her life as a new mother living with arthritis, she encountered challenges with both her mental and physical health that she felt could have been better supported. Her experience has given us new perspectives on advice for expectant mothers with arthritis: 1) begin advocating for family planning conversations early with rheumatologists, 2) reach out to other pregnancy and arthritis community members for support, and to 3) access essential services in mental health, arthritis management and occupational therapy throughout pregnancy and postpartum.

Another significant layer to navigating motherhood with arthritis is the undeniable health inequities and longstanding history of abuse from the medical community towards Black individuals. Due to the overrepresentation of Black individuals in the child welfare system and their disproportionate adverse outcomes, we learned that Black mothers with arthritis may be hesitant to seek support, fearing they may be misperceived as incapable of caring for their child. This highlights the need to collectively provide compassionate, culturally sensitive care to all individuals, while increasing practitioner education on disease management in Black patients. Importantly, the disproportionate adverse pregnancy outcomes and systemic disparities faced by Black women in Canada are underexamined and require more action-oriented research to increase awareness, acknowledgement and resolution of systemic issues.

As revealed by our patient speakers, women living with arthritis who intend to conceive are eager to seek out family planning services, but often face difficulties in finding specialized care. For this reason, the first Pregnancy and Rheumatic Diseases Clinic was established in British Columbia, Canada. This clinic is an incredible example of a novel model of arthritis care that is dedicated to offering specialized counseling services prior to and during pregnancy, where rheumatologists carefully address family planning concerns and conduct medication reviews to ensure optimal maternal–fetal safety. We gathered invaluable insights from the perspectives of a rheumatologist, which mirrored the sentiments shared by our patient speakers. Mainly, patients are strongly encouraged to engage in early, individualized family planning conversations with their rheumatologist regarding the risks and benefits of treatment options. We learned that patients who received pre-pregnancy counseling were less likely to experience flares, undergo medication changes during pregnancy, and more likely to have lower disease activity in first trimester—all indicative of positive pregnancy outcomes [2]. Accordingly, we learned it is recommended for rheumatologists to refer patients for pre-pregnancy consultation 1–2 years prior to conception.

The Pregnancy and Arthritis webinar was concluded on an encouraging note from the perspective of our researchers. We were taken through a timeline of early research on arthritis in pregnancy beginning from the early 2000’s, which marked the emergence of biologics. Made possible through collaborations with patient partners and clinicians, we now have a better understanding of the perinatal impacts of many arthritis medications on both mother and baby, along with complexities involved in decision-making for mothers and their partners. As newer therapies are introduced and longer-term data becomes available, research is continuing to expand. In hopes of filling the current gaps in knowledge on the perinatal impacts of emerging biosimilars and targeted therapies, our webinar concluded with the hopeful reassurance that ongoing research is underway and could be expanded to include an interdisciplinary approach to healthcare.

### Perspectives shared on parenting and arthritis

The subsequent webinar on Parenting and Arthritis also integrated multiple perspectives and experiences from a researcher and a clinician/researcher, which triangulated in the gathering of evidence-based resources and strategies to support those parenting with arthritis.

With respect to supports, the webinar highlighted Indigenous doulas who: 1) offer comprehensive care before, during, post-birth and during other reproductive life events, 2) address physical and spiritual health, and 3) play important roles in kinship and community life. A 2021 qualitative study showed that Indigenous doula care addresses a wide range of issues that affect Indigenous women’s experiences across pregnancy, birth, and post-partum [3]. This is important given that Indigenous women are disproportionately affected by arthritis and experience poorer maternal outcomes. We also learned that ongoing research at the University of Manitoba will develop and evaluate an urban Indigenous doula curriculum in Winnipeg.

The intersection of “doing [parenting]” (“*patients with arthritis report more difficulties with parenting tasks than individuals without arthritis*”) and “being [a parent]” (“*parents with arthritis report similar sense of competency and satisfaction (joy) being parents as those without arthritis”*) was then explored, particularly using evidence-based strategies to support parenting with arthritis. Qualitative research by McDonald et al. highlighted “doing things differently” and “changing views of self” as ways that patients addressed the impact of their rheumatoid arthritis on parenting roles, tasks, and activities [4]. Occupational balance—the subjective experience of having the right amount and variety of activities (including paid and unpaid work, leisure, rest, and sleep)—was introduced as an approach to leading a more satisfying life amidst competing demands [5]. Practical advice to support parenting with arthritis included: 1) pacing oneself (e.g., moderation); 2) prioritizing (e.g., taking ownership of health); 3) planning; and 4) positioning (e.g., ergonomics to support body while parenting). Advice also included changing perceptions of activity, such as inserting physical activity throughout the day or week in minutes (versus hours). Lastly, we learned the importance of delegating tasks and seeking family, friends, and professionals, particularly occupational therapists, who can provide support for parenting activities.

## Conclusion

These public webinars brought together diverse perspectives from researchers, patients, and clinicians to discuss and raise awareness relating to pregnancy and parenting with arthritis. Dismantling the long-held notion that arthritis is an “old person’s disease”, these webinars highlighted the profound impacts of arthritis on patients and families. Along with bringing awareness to these impacts, discussions during the webinars also revealed knowledge gaps for future research. There is a need to continue generating evidence on perinatal impacts of arthritis medications, developing and evaluating novel models of care for arthritis patients who are pregnant or planning to become pregnant, combating health inequities particularly among Black and Indigenous individuals, as well as addressing the support needs of arthritis patients and their families across the lifespan.

## Data Availability

Not applicable.

## Abbreviations

CAPA: Canadian Arthritis Patient Alliance
UBC: University of British Columbia
